# Assessment of antimicrobial resistance laboratory-based surveillance capacity of hospitals in Zambia: findings and implications for system strengthening

**DOI:** 10.1016/j.jhin.2024.03.014

**Published:** 2024-06

**Authors:** K. Yamba, J.Y. Chizimu, S. Mudenda, C. Lukwesa, R. Chanda, R. Nakazwe, B. Simunyola, M. Shawa, A.C. Kalungia, D. Chanda, T. Mateele, J. Thapa, K. Kapolowe, M.L. Mazaba, M. Mpundu, F. Masaninga, K. Azam, C. Nakajima, Y. Suzuki, N.N. Bakyaita, E. Wesangula, M. Matu, R. Chilengi

**Affiliations:** a Antimicrobial Resistance Coordinating Committee Unit, Zambia National Public Health Institute, Lusaka, Zambia; b Department of Pharmacy, School of Health Sciences, University of Zambia, Lusaka, Zambia; c Department of Health, Lusaka District Health Office, Lusaka, Zambia; d Department of Pathology and Microbiology, University Teaching Hospitals, Lusaka, Zambia; e Department of Pharmacy, Ministry of Health, Lusaka, Zambia; f Hokudai Center for Zoonosis Control in Zambia, Hokkaido University International Institute for Zoonosis Control, Lusaka, Zambia; g Department of Internal Medicine, University Teaching Hospitals, Lusaka, Zambia; h Department of Internal Medicine, Levy Mwanawasa University Teaching Hospital, Lusaka, Zambia; j Division of Bioresources, Hokkaido University International Institute for Zoonosis Control, Sapporo, Hokkaido, Japan; k Action on Antibiotic Resistance (ReAct) Africa, Lusaka, Zambia; l Department of Health, World Health Organization, Lusaka, Zambia; m Strengthening Pandemic Preparedness, Eastern and Southern Africa Health Community, Arusha, Tanzania; n International Collaboration Unit, Hokkaido University International Institute for Zoonosis Control, Sapporo, Hokkaido, Japan; o Division of Research Support, Hokkaido University Institute for Vaccine Research and Development, Sapporo, Hokkaido, Japan

**Keywords:** Antimicrobial resistance, Antimicrobial stewardship, Laboratory capacity, Surveillance, Zambia

## Abstract

**Background:**

A well-established antimicrobial resistance (AMR) laboratory-based surveillance (LBS) is of utmost importance in a country like Zambia which bears a significant proportion of the world's communicable disease burden. This study assessed the capacity of laboratories in selected hospitals to conduct AMR surveillance in Zambia.

**Methods:**

This cross-sectional exploratory study was conducted among eight purposively selected hospitals in Zambia between August 2023 and December 2023. Data were collected using the self-scoring Laboratory Assessment of Antibiotic Resistance Testing Capacity (LAARC) tool.

**Findings:**

Of the assessed facilities, none had full capacity to conduct AMR surveillance with varying capacities ranging from moderate (63% (5/8)) to low (38% (3/8)). Some of the barriers of AMR-LBS were the lack of an electronic laboratory information system (63% (5/8)) and the lack of locally generated antibiograms (75% (6/8)). Quality control for antimicrobial susceptibility testing (AST), pathogen identification and media preparation had the lowest overall score among all of the facilities with a score of 14%, 20% and 44%, respectively. The highest overall scores were in specimen processing (79%), data management (78%), specimen collection, transport and management (71%), and safety (70%). Most facilities had standard operating procedures in place but lacked specimen-specific standard operating procedures.

**Conclusion:**

The absence of laboratories with full capacity to conduct AMR surveillance hinders efforts to combat AMR and further complicates the treatment outcomes of infectious diseases. Establishing and strengthening LBS systems are essential in quantifying the burden of AMR and supporting the development of local antibiograms and treatment guidelines.

## Introduction

Laboratory-based surveillance is essential in monitoring antimicrobial resistance (AMR) [1,2]. AMR is a global threat of public health concern that needs to be controlled as it poses a serious threat to the treatment of infectious diseases [[3], [4], [5]]. AMR occurs when bacteria, viruses, fungi and parasites mutate and acquire the ability to defeat the effects of antimicrobials that were previously effective [6]. This makes treating diseases more challenging and increases the risk of disease spread, significant illness and death [7,8]. Due to this burden of AMR, laboratories should be strengthened and capacitated to conduct AMR surveillance [9,10].

Of the global death rates reported in 2019, approximately 24/100,000 were from sub-Saharan Africa [7]. The region has high incidences of infectious diseases such as human immunodeficiency virus/acquired immunodeficiency syndrome (HIV/AIDS), malaria and tuberculosis [[11], [12], [13], [14], [15], [16], [17]]. Therefore, high trends of AMR to the agents causing infections in resource-constrained regions further weaken the already stressed health systems [18]. Though AMR can occur as a natural process as a way of adaptation of the pathogen to its environment, the misuse and overuse of antimicrobials have contributed to the rise of AMR [[19], [20], [21]].

Addressing AMR requires coordinated multi-faceted approaches [[22], [23], [24]]. To this effect, a Global Action Plan (GAP) on AMR was developed in 2015 to address drug-resistant infections [24]. Strategies including antimicrobial stewardship (AMS) are critical in the fight against AMR [[25], [26], [27], [28], [29], [30], [31]]. The GAP emphasizes the need for developing and implementing AMS programmes in hospitals [24]. Further, it promotes the need to strengthen AMR education and awareness campaigns, and advocates for more innovative research to develop strategies that will address AMR [24]. Subsequently, national authorities and local hospitals should develop AMR surveillance systems that are essential for monitoring evolving trends in AMR [[32], [33], [34]]. Laboratories are a critical component of AMS and AMR surveillance and guide the best treatment options for a particular pathogen based on the surveillance data [[35], [36], [37], [38]].

Zambia, one of the countries in sub-Saharan Africa, has reported the rising trends in AMR to the commonly used antibiotics in selected publications [34,[39], [40], [41], [42], [43], [44], [45]]. Some of the risk factors contributing to the emergence and spread of AMR in Zambia include a lack of diagnostic services, poor infection prevention and control in hospitals, inappropriate use of antibiotics in hospitals, communities, veterinary, agriculture, and the environment, a lack of updated evidence-based treatment guidelines based on antibiotic use, and a lack of enforcement of laws and regulations on appropriate use of antibiotics to curb AMR [42,[46], [47], [48], [49]]. Poor quality assurance and lack of laboratory consumables have contributed to a low capacity of laboratories to conduct AMR surveillance in Zambia [46]. Several interventions enshrined in the Zambia Multi-sectoral National Action Plan (NAP) on AMR have been instituted to curb the development of AMR in healthcare facilities in Zambia [45,50], including training of healthcare workers in AMR and AMS, creation of awareness among community members, development of treatment and AMS guidelines. The laboratory is one of the key pillars in combating the emergence of AMR as it provides evidence of resistance through testing and analysis of micro-organisms to determine their susceptibility or resistance to various antimicrobial agents. Intriguingly, laboratories guide clinicians on the better management of patients and provide information for infection prevention and control and diagnostic surveillance for AMS mitigation [51,52]. Nevertheless, like most developing countries in Africa [9,46,53], there is inadequate information in Zambia on the capacity of the local laboratories to conduct microbiological tests required for AMR surveillance.

Therefore, this study assessed the laboratory capacity to conduct AMR surveillance in selected secondary and tertiary hospitals in Zambia. The assessment offers a starting point for understanding the strengths and weaknesses and provides crucial information for developing effective strategies to improve the laboratories' diagnostic capacity to address the issue of AMR.

## Materials and methods

### Study design and sites

A cross-sectional exploratory study was conducted in eight public hospitals across Zambia to assess the capacity of the local laboratories for AMR surveillance between August and December 2023. The hospital facilities, namely, Arthur Davison Children's Hospital (ADH) in the Copperbelt Province, Chilonga Mission Hospital (CMH) in the Northern Province, Chipata Central Hospital (CCH) in the Eastern Province, Kabwe General Hospital (KGH) in the Central Province, Kitwe Teaching Hospital (KTH) in the Copperbelt Province, Livingstone Central Hospital (LCH) in the Southern Province, Mansa General Hospital (MGH) in the Luapula Province, and Ndola Teaching Hospital (NTH) in the Copperbelt Province, respectively, were selected from six of the 10 provinces of Zambia.

### Sample size and sampling criteria

A total of eight public hospitals constituted the sample size out of which five were tertiary-level hospitals (i.e., ADH, CCH, KTH, LCH and NTH) and three were secondary level hospitals (i.e., CMH, KGH and MGH). The selection of the hospital laboratories included in the assessment was based on their ability to provide minimum to advanced laboratory testing at their levels of health care provision in Zambia. The eight facilities were purposely chosen to provide baseline data on the laboratory capacities to generate AMR testing data. From each facility, three laboratory personnel were purposely selected because they were working in the laboratory section of the facility. We excluded clinics because they were not at the level of conducting bacteriology in their laboratories.

### Data collection

The assessment involved interviews, inspection of the laboratories and laboratory processes, as well as verification of documents. This primary data collection tool was the Laboratory Assessment of Antibiotic Resistance Testing Capacity (LAARC) version 2.0 of 2020, developed by the Centers for Disease Control and Prevention (CDC) [54]. It is a laboratory assessment tool for use in clinical bacteriology laboratories in low- and middle-income countries. The tool contains 14 core indicators including: facility; laboratory information system (LIS); data management; quality assurance; quality control (media); quality control (identification); quality control (antimicrobial susceptibility testing, AST); specimen collection, transportation, and management; sample processing; identification methods, and standard operating procedures (SOPs); basic AST, AST expert rules; AST panel, policy, and analysis; and safety. For each facility, at least three laboratory staff were interviewed, evidence checked and recorded in the LAARC self-scoring tool. The findings were used to generate facility-specific strengths, weaknesses, opportunities and challenges.

### Data analysis

The LAARC tool provided a summary of the percentage scores of the 14 core indicators. The summaries were then aggregated on the heat map to show each facility's scores in each of the core indicators. Based on the CDC LAARC tool, scores of 0–49% were considered below average (low capacity) whereas those between 50 and 79% were considered average (moderate capacity) [54]. Finally, scores of 80% and above were considered good (full capacity) [54]. The other data consisting of open-ended responses from laboratory personnel interviewed at each participating hospital were analysed using thematic analysis and presented as narratives.

### Ethical approval

Ethical approval was granted by the Tropical Diseases Research Centre (TDRC) Ethics Committee with an approval number of TRC/C4/09/2023. The purpose of this study was communicated to the hospital management and the participants. Confidentiality and anonymity were maintained throughout the study.

## Results

**Figure 1:**
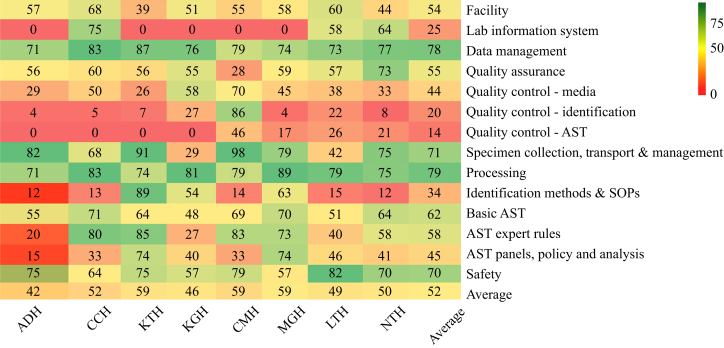
Baseline capacities of laboratories to conduct surveillance AMR in eight hospitals. ADH, Arthur Davison Children's Hospital; AST, antimicrobial susceptibility testing; CCH, Chipata Central Hospital; CMH, Chilonga Mission Hospital; KGH, Kabwe General Hospital; KTH, Kitwe Teaching Hospital; LTH, Livingstone Teaching Hospital; MGH, Mansa General Hospital; NTH, Ndola Teaching Hospital; SOPs, standard operating procedures.

Overall, of the eight facilities, five (62.5%) had moderate capacity to implement AMR surveillance whereas three (37.5%) had low capacity. None of the surveyed hospitals had the full capacity to implement AMR surveillance. Further, below-average scores (low capacity) were observed with some indicators including quality control (14%), quality control identification (20%), LIS (25%), identification methods and SOPs (34%), quality control of media (44%), AST panels, and policy and analysis (45%). However, facilities scored moderately in eight of 14 indicators (57%) with the highest being specimen processing (79%), data management (78%), specimen collection, transport, management (71%), safety (70%), and basic AST (62%), and lowest in AST expert rules (58%), quality assurance (55%), and facility (54%). An overall capacity of 52% was recorded (Figure 1).

### Strengths and gaps in the facility and laboratory information system indicators among the different hospitals

All eight hospital facilities (100%) assessed for AMR surveillance laboratory capacity had laboratory infrastructure in place, however, the facilities' scores varied, with an average score of 52%. NTH, which scored the lowest (44%) on the facility assessment, had some gaps ranging from the lack of a contingency plan in the event of prolonged electricity disruption, and the lack of -20 °C and -80 °C freezers for prolonged storage and preservation of isolates. CCH, which had the highest score (68%) under the facility assessment, adhered to quality assurance indicators such as documentation of temperature and atmosphere monitoring and had automated diagnostic equipment that was under annual maintenance (Supplementary data).

Only three (38%) facilities (CCH, NTH and LTH) had an electronic LISs in place. Data management was above 70% in all of the health facilities, including those that did not have a LIS in place and used paper-based information systems. It was observed that both LISs and paper-based data comprised the most critical information needed for clinical sample processing with very few variables missing (Supplementary material). The critical variables included patient demographics, date of admission, and patient location (ward of admission) but lacked information that helps differentiate community-acquired infections (CAIs) from hospital-acquired infections (HAIs). Further, the LIS had no provision to enter qualitative AST results such as disc diffusion and minimum inhibitory concentration (MIC) values and lacked the software to automatically interpret AST results (Supplementary material).

### Strengths and gaps in quality assurance and management of data among AST facilities

All eight laboratories had a quality management system and safety measures in place, most of which continue to require improvement to improve accurate diagnosis and qualify for accreditation. All of the facilities had quality manuals in place and participated in external quality assurance (EQA), however, most facilities had unacceptable EQA/proficiency testing results below the recommended target of >80%. It was notable that most of the laboratories did not conform to the quality control processes for media preparation, pathogen identification and AST. Six of the eight (75%) laboratories scored high in specimen collection, transport and management with only KGH (29%) and LTH (42%) scoring low in these particular indicators (Supplementary material). Almost all of the laboratories had general SOPs but lacked specimen-specific processing SOPs. Only MGH and NTH had antibiograms based on their locally generated microbiology data while the other hospitals either lacked the capacity or had inadequate data to generate antibiograms. All of the assessed facilities reported challenges related to frequently interrupted supply (stock-outs) of laboratory consumables and reagents necessary for culture and AST (Supplementary material). Noteworthy was the presence of non-functional equipment and poor non-compliance to preventive maintenance measures.

### Strengths and gaps in quality control for media, identification and AST

Quality control for AST, identification and media had the lowest overall score among all of the hospital facilities with a score of 14%, 20% and 44%, respectively. All of the facilities except for CMH scored below 50% for quality control of identification and AST. The facilities that had non-existent (0%) quality control for AST and low scores (<10%) in quality control measures in the identification of pathogens had no ATCC (American Type Culture Collection) reference strains to quality control antibiotics, biochemical tests, and commercial and automated identification systems where applicable (Supplementary material). The low scores in quality control for media preparation were attributed to a lack of media preparation SOPs, a lack of separate sterile rooms away from specimen processing rooms, and the lack of availability of sheep blood for the preparation of blood agar media. Only CMH was found to use ATCC reference strains to quality control for identification and media preparation, all of which were documented in line with the quality management system requirements, hence their high score of 86% and 70%, respectively, in these particular indicators (Supplementary material).

### Strengths and gaps in AST-basic, AST expert rules, AST panels, policy and analysis

All of the facilities scored above 50% for basic AST (overall 62%) and AST expert rules (overall 58%) except for KGH which scored 48% and 27%, respectively (Supplementary material). The facility scored 0% on breakpoint standards and Gram-negative/beta-lactam break points. The overall score for AST panels, policy and analysis was 48%. This was due to a lack of AST panels at ADH, CCH, LTH and CMH as well as a lack of cumulative antibiograms at ADH, CCH, KGH and KTH (Supplementary material).

### Strengths and gaps in specimen collection, transport, management and laboratory safety

All of the facilities scored above 50% for specimen collection, transport and management (overall score of 71%) except for KGH and LTH which scored 29% and 42%, respectively. KGH scored 0% on specimen collection and transport for all sample types (blood, urine and stool) whereas LTH scored 15% on specimen collection and transport for blood samples (Supplementary material). The low scores were a result of the laboratory not having a back-up arrangement with another laboratory to process urgent specimens or store them at the proper temperature if the laboratory was closed. Further, the laboratories lacked specimen rejection criteria in their SOPs and bench aids and did not provide specimen collection instructions or SOPs for the clinical/collection areas. For the safety indicator, all of the facilities scored way above 50% except for KGH and MGH which scored 57% each. The overall score for the safety indicator was 70%. Both facilities scored poorly (30%) on biosafety equipment (Supplementary material).

### Equipment availability

The availability of equipment in the assessed facilities varied with most laboratories lacking equipment. Only one laboratory had the McFarland standard that is used as a reference to adjust the turbidity of bacterial suspension and none of the laboratories had a Turbidimeter for determining McFarland density. None of the laboratories had carbon dioxide incubators with only four laboratories using candle jars (Supplementary material). Only one laboratory had a biological safety cabinet class IIA. Four laboratories had 2–8 °C refrigerators, three laboratories had -20 °C freezers, while none had -60 °C and -80 °C freezers. Three laboratories documented having calibrated loops for urine culture and only two laboratories had autoclaves for sterilizing waste. In terms of automated equipment for identification and AST, four laboratories had functional automated blood culture systems while none of the laboratories had automated identification and AST equipment such as Vitek, Phoenix, matrix-assisted laser desorption/ionization (MALDI-TOF) and polymerase chain reaction for AMR gene detection (Supplementary material).

This study found that most selected hospitals had challenges including inadequate funding, stock-out of laboratory consumables, lack of training of healthcare workers on AMR and AMS, limited capacity to develop antibiograms, and a lack of automated equipment for pathogen identification and susceptibility testing (Table I).

**Table I:** Barriers to the implementation of laboratory-based surveillance of antimicrobial resistance (AMR)

Barrier number	Barriers towards implementation of AMR surveillance
i.	Inadequate funding
ii.	Stock-outs of laboratory consumables
iii.	Interruption of service delivery
iv.	Inconsistent use of guidelines for interpretation of AST results
v.	Using paper-based LIS
vi.	Lack of training of healthcare workers on AMR and AMS
vii.	Limited capacity to develop antibiograms
viii.	Inconsistent quality control and lack of ATCC control strains
ix.	Lack of standard operation procedures
x.	Lack of automated equipment for pathogen identification and susceptibility testing

AMS, antimicrobial stewardship; AST, antimicrobial susceptibility testing; ATCC, American Type Culture Collection; LIS, laboratory information system.

## Discussion

This study assessed the capacity of local laboratories to conduct AMR surveillance in selected hospitals in Zambia. Despite all hospitals assessed in this study having laboratory infrastructure, none of the facilities had full capacity to conduct AMR surveillance due to a lack of trained microbiology staff, financial challenges, stock-outs of laboratory consumables, use of paper-based LISs, lack of training in AMR and AMS, lack of antibiograms, lack of automated equipment for pathogen identification and susceptibility testing, and poor implementation of safety and biosecurity measures.

This study found that all hospitals that were assessed had laboratory infrastructure, a critical key component of AMR surveillance [55]. Similarly, a study in Ethiopia found that all healthcare facility laboratories surveyed had laboratory facilities [53]. In our study, however, an overall capacity of 52% to implement AMR surveillance was noted, with the lowest capacity being at ADH and the highest being at KTH, CMH and MGH, respectively. Despite not being highly considered in the scoring of all indicators, our study found that the lack of a LIS was one of the contributing factors to the poor score in the laboratory capacity to conduct AMR surveillance. Consequently, the presence of a LIS in facilities not only elevates the capacities of the clinical laboratories but also reduces the diagnostic errors, and the time required for reporting results and improves data quality and analysis thus enhancing the decision-making process and leading to better treatment and diagnostic outcomes [56,57]. Deficiencies in quality control for pathogen identification and ASTs, antibiograms, trained microbiology staff, supplies of laboratory consumables, and implementation of safety and biosecurity measures may result in unfavourable treatment outcomes, irrational use of antimicrobials and increased risk of transmission of drug-resistant infections [[58], [59], [60]].

Our finding that none of the surveyed local laboratories had the full capacity to effectively conduct AMR surveillance was a cause for concern given the important roles that laboratories play to address this problem. However, most facilities (five of eight) were found to have moderate capacity implying a potential for improvement and development with dedicated support. These findings were in line with a previous study conducted across Kenya, Tanzania, Uganda and Zambia [46] and another study carried out in Ghana [1]. A study by Moirongo and colleagues in Kenya reported inadequate capacities among laboratories specifically in the areas of LISs, external quality assurance, laboratory supplies and equipment [9]. The 2021 report on the surveillance of AMR in low- and middle-income countries highlighted the barriers to effective surveillance in AMR as weak laboratory infrastructure, limited staff capacity and training, poor communication between laboratory staff and clinical teams, questionable quality assurance and limited availability of diagnostic consumables and reagents, as a result of heavy reliance on external funding [61]. The inadequacy of laboratory capacities affects their functionality in pathogen identification and thereby overall AMR surveillance [35,62]. Addressing these barriers could lead to improved AMR surveillance.

Notwithstanding that the overall score for quality control for media and AST were both below average, the quality control of media used in clinical microbiology laboratories remains critical for accurate and acceptable isolation of pathogens from infected patients. Poor quality control of media preparation may, therefore, compromise the microbiology results [63]. Erroneous results hinder the possibility of accurate treatment and the management of infectious diseases, thereby negatively affecting treatment outcomes [64]. Interpretation of AST results should be based on international guidelines for testing and interpretation, such as the Clinical and Laboratory Standards Institute (CLSI), however, most of the facilities in our study performed poorly in quality control for AST testing, which could result in unreliable surveillance data being used for the development of antibiograms and policy-driven interventions [65,66].

Our study further found that only two hospital facilities had antibiograms developed from locally generated data, implying that there is widespread use of empirical treatment without definitive susceptibility results in the majority of hospitals in Zambia, a practice that promotes the emergence of AMR [67]. The lack of antibiograms in most hospitals in this study could be attributed to the absence of LISs resulting in manual entry of microbiology data and the frequent stock-outs of laboratory reagents and consumables coupled with limited expertise among laboratory staff in interpreting and formulating antibiograms [68]. Further, the lack of antibiograms in some hospitals affects the surveillance of AMR [69,70]. A study in Ghana equally found inadequate availability of cumulative antibiogram data for bacterial pathogens to aid specific interventions that augment AMS implementation [71]. These challenges are very common in low- and middle-income countries due to limited resources to support these critical activities [61]. Antibiograms are valuable tools for guidance of clinical decisions, epidemiological surveillance of emerging resistance trends, and guiding empiric therapy for AMS support. In Uganda, it was found that the unavailability of antimicrobials for AST affected the capacity of laboratories and made AMR surveillance difficult [68].

The absence of the McFarland standard, the reference used to adjust bacterial suspension for AST in all but one laboratory and the absence of turbidimeters in all of the laboratories in our study is an indication that AST was being carried out without following recommended guidelines. This practice would provide incorrect AST results and would be misleading in the treatment of infectious diseases [66,72]. Accurate and rapid detection of antimicrobial susceptibility and subsequent appropriate antimicrobial treatment, combined with AMS, are essential for controlling the emergence and spread of AMR [72].

The absence of carbon dioxide incubators in all of the surveyed laboratories compromises the ability to isolate fastidious organisms of public health significance such as *Streptococcus pneumoniae*, *Haemophilus influenzae* and *Neisseria meningitidis*, thereby limiting the hospital's ability to correctly treat patients infected with these organisms and monitor the changing trends, data that can support country- and/or region-specific vaccine development [73,74]. Further, the lack of -60 °C and -80 °C freezers in these facilities hinders the long-term storage of bacterial isolates that can be used for future analysis and surveillance. Lastly, the absence of automated identification and AST equipment in all of the assessed facilities is of great concern as automated identification and AST equipment not only provide accurate diagnosis but also enable accurate and timely treatment thereby improving treatment outcomes and reducing the irrational use of antimicrobials [75].

Training healthcare workers in AMR surveillance and providing adequate resources to the facilities would help address the reported gaps and incapacities in our study. Providing these strategies would scale up the capacities of laboratories and human resources in the surveillance of AMR. Such findings have been reported in other studies where interventions led to better outcomes such as increased performance of ASTs and reduction in antibiotic use [25].

Building on the findings of this study, improving the capacity of local hospital laboratories in Zambia for AMR surveillance must involve a multi-faceted strategy. We contend that it is crucial to invest in upgrading infrastructure and providing modern equipment while ensuring a stable supply of essential laboratory consumables. Additionally, a focus on training programmes and capacity-building for laboratory staff in AMR surveillance techniques is essential. Quality assurance measures, including proficiency testing, should be implemented to guarantee the accuracy and reliability of laboratory results. Developing a robust data management system, standardized protocols, and fostering collaboration among stakeholders is pivotal for efficient data collection and analysis. Moreover, strengthening regulatory frameworks, supporting research and innovation, and engaging in international collaboration for best practices further contribute to enhancing local laboratory capacities in conducting effective AMR surveillance in Zambia.

We are aware of some limitations of this study. Our study surveyed a limited number of local laboratories at selected hospital facilities, and the findings may not be generalizable to all hospitals across the public and private hospitals with different demographics and settings in Zambia. Consequently, generalization of the findings should be carried out with caution and only performed in hospital settings similar to the surveyed hospitals. We are also concerned that our findings may have bene affected by the COVID-19 pandemic. Notwithstanding this, we ensured that the hospitals selected in this study were representative of the types and nature of tertiary and secondary hospital facilities that are found across Zambia. We, therefore, remain confident that the findings may not be significantly different in other similar hospitals across the country.

In conclusion, this study found that none of the surveyed hospitals had the full capacity to effectively conduct AMR surveillance. Most of the facilities had moderate and low capacity to implement laboratory-based AMR surveillance. The absence of laboratories with full capacity to conduct AMR surveillance hinders efforts to combat AMR and further complicates the treatment outcomes of infectious diseases. Establishing and strengthening laboratory-based surveillance systems are essential in quantifying the burden of AMR and supporting the development of local antibiograms and treatment guidelines. To make gains in mitigating AMR spread, there is, therefore, an urgent need to address the identified barriers and improve laboratory-based surveillance of AMR as a critical component of AMS. The findings of this study serve as a starting point to strengthen laboratory-based surveillance in line with the national and global AMR action plans.

## Author contributions

K.Y., J.Y.C., S.M., E.W. and R.C. contributed to the initial study concept. K.Y., J.Y.C., S.M., C.L., R.C., R.N., B.S., D.C., T.M., M.L.M., M.M., K.A., E.W., M.M. and R.C. contributed to the study design. J.Y.C. and S.M. oversaw all study activities. K.Y., J.Y.C., S.M., C.L., R.N., B.S. and T.M. participated in the acquisition of the data. K.Y., A.C.K., K.K., F.M. and N.N.B. advised on all qualitative aspects of the study. K.Y., J.Y.C., S.M., C.L., R.N., F.M., K.A., C.N., N.N.B., E.W., M.M. and S.Y. interpreted the data. K.Y., J.Y.C., S.M., R.N., T.M., K.K. and M.L.M. wrote initial drafts. B.S., T.M., M.L.M., A.C.K., M.M. and R.C. critically revised the article for important intellectual content. K.Y., J.Y.C., S.M., C.N. and S.Y. performed the quantitative and qualitative data analysis. All authors contributed to the final editing of the article. All authors approved the final version of the article to be published.

## Conflict of interest statement

The authors declare that they have no known competing financial interests or personal relationships that could have appeared to influence the work reported in this paper.

## Funding sources

This work was supported by the World Bank funding, Strengthening Pandemic Preparedness Project, through Eastern, Central and Southern Africa Health Community, (ECSA-HC), the World Health Organization, and in part by the Japan Agency for Medical Research and Development (10.13039/100009619AMED) (Grant Number JP23wm0125008 and JP233fa627005 to Y.S).

## References

[1] Opintan J.A., Newman M.J., Arhin R.E., Donkor E.S., Gyansa-Lutterodt M., Mills-Pappoe W. (2015). Laboratory-based nationwide surveillance of antimicrobial resistance in Ghana. Infect Drug Resist.

[2] Vounba P., Loul S., Tamadea L.F., Siawaya J.F.D. (2022). Microbiology laboratories involved in disease and antimicrobial resistance surveillance: strengths and challenges of the central African states. Afr J Lab Med.

[3] Walsh T.R., Gales A.C., Laxminarayan R., Dodd P.C. (2023). Antimicrobial resistance: Addressing a global threat to humanity. PLoS Med.

[4] Acharya K.P., Wilson R.T. (2019). Antimicrobial resistance in Nepal. Front Med.

[5] Hinchliffe S., Butcher A., Rahman M.M. (2018). The AMR problem: demanding economies, biological margins, and co-producing alternative strategies. Palgrave Commun.

[6] Prestinaci F., Pezzotti P., Pantosti A. (2015). Antimicrobial resistance: a global multifaceted phenomenon. Pathog Glob Health.

[7] Murray C.J., Ikuta K.S., Sharara F., Swetschinski L., Robles Aguilar G., Gray A. (2022). Global burden of bacterial antimicrobial resistance in 2019: a systematic analysis. Lancet.

[8] Ikuta K.S., Swetschinski L.R., Robles Aguilar G., Sharara F., Mestrovic T., Gray A.P. (2022). Global mortality associated with 33 bacterial pathogens in 2019: a systematic analysis for the Global Burden of Disease Study 2019. Lancet.

[9] Moirongo R.M., Aglanu L.M., Lamshöft M., Adero B.O., Yator S., Anyona S. (2022). Laboratory-based surveillance of antimicrobial resistance in regions of Kenya: an assessment of capacities, practices, and barriers by means of multi-facility survey. Front Public Health.

[10] Bertagnolio S., Suthar A.B., Tosas O., Van Weezenbeek K. (2023). Antimicrobial resistance: strengthening surveillance for public health action. PLoS Med.

[11] Chizimu J.Y., Solo E.S., Bwalya P., Kapalamula T.F., Mwale K.K., Squarre D. (2023). Genomic analysis of mycobacterium tuberculosis strains resistant to second-line anti-tuberculosis drugs in Lusaka, Zambia. Antibiotics.

[12] Mbewe N., Vinikoor M.J., Fwoloshi S., Mwitumwa M., Lakhi S., Sivile S. (2022). Advanced HIV disease management practices within inpatient medicine units at a referral hospital in Zambia: a retrospective chart review. AIDS Res Ther.

[13] Formenti B., Gregori N., Crosato V., Marchese V., Tomasoni L.R., Castelli F. (2022). The impact of COVID-19 on communicable and non-communicable diseases in Africa: a narrative review. Infez Med.

[14] Margolin E., Burgers W.A., Sturrock E.D., Mendelson M., Chapman R., Douglass N. (2020). Prospects for SARS-CoV-2 diagnostics, therapeutics and vaccines in Africa. Nat Rev Microbiol.

[15] Monroe A., Williams N.A., Ogoma S., Karema C., Okumu F. (2022). Reflections on the 2021 World Malaria Report and the future of malaria control. Malar J.

[16] Mweemba C., Hangoma P., Fwemba I., Mutale W., Masiye F. (2022). Estimating district HIV prevalence in Zambia using small-area estimation methods (SAE). Popul Health Metr.

[17] Bhutta Z.A., Sommerfeld J., Lassi Z.S., Salam R.A., Das J.K. (2014). Global burden, distribution, and interventions for infectious diseases of poverty. Infect Dis Poverty.

[18] Gulumbe B.H., Haruna U.A., Almazan J., Ibrahim I.H., Faggo A.A., Bazata A.Y. (2022). Combating the menace of antimicrobial resistance in Africa: a review on stewardship, surveillance and diagnostic strategies. Biol Proced Online.

[19] Amann S., Neef K., Kohl S. (2019). Antimicrobial resistance (AMR). Eur J Hosp Pharm.

[20] Harbarth S., Balkhy H.H., Goossens H., Jarlier V., Kluytmans J., Laxminarayan R. (2015). Antimicrobial resistance: one world, one fight. Antimicrob Resist Infect Control.

[21] Kahn L.H. (2017). Antimicrobial resistance: a One Health perspective. Trans R Soc Trop Med Hyg.

[22] Joshi M.P., Hafner T., Twesigye G., Ndiaye A., Kiggundu R., Mekonnen N. (2021). Strengthening multisectoral coordination on antimicrobial resistance: a landscape analysis of efforts in 11 countries. J Pharm Policy Pract.

[23] Mudenda S., Chabalenge B., Daka V., Mfune R.L., Salachi K.I., Mohamed S. (2023). Global strategies to combat antimicrobial resistance: One Health perspective. Pharmacol Pharm.

[24] World Health Organization (2015). https://apps.who.int/iris/handle/10665/193736.

[25] Kwabena O., Amponsah O., Courtenay A., Kwame Ayisi-Boateng N., Abuelhana A., Opoku D.A. (2023). Assessing the impact of antimicrobial stewardship implementation at a district hospital in Ghana using a health partnership model. JAC Antimicrob Resist.

[26] Godman B., Egwuenu A., Haque M., Malande O.O., Schellack N., Kumar S. (2021). Strategies to improve antimicrobial utilization with a special focus on developing countries. Life.

[27] Majumder M.A.A., Rahman S., Cohall D., Bharatha A., Singh K., Haque M. (2020). Antimicrobial stewardship: fighting antimicrobial resistance and protecting global public health. Infect Drug Resist.

[28] Al-Omari A., Al Mutair A., Alhumaid S., Salih S., Alanazi A., Albarsan H. (2020). The impact of antimicrobial stewardship program implementation at four tertiary private hospitals: results of a five-year pre-post analysis. Antimicrob Resist Infect Control.

[29] Siachalinga L., Mufwambi W., Lee I.-H. (2022). Impact of antimicrobial stewardship interventions to improve antibiotic prescribing for hospital inpatients in Africa: a systematic review and meta-analysis. J Hosp Infect.

[30] Mendelson M., Morris A.M., Thursky K., Pulcini C. (2020). How to start an antimicrobial stewardship programme in a hospital. Clin Microbiol Infect.

[31] Saleem Z., Godman B., Cook A., Khan M.A., Campbell S.M., Seaton R.A. (2022). Ongoing efforts to improve antimicrobial utilization in hospitals among African countries and implications for the future. Antibiotics.

[32] World Health Organization (2022).

[33] World Health Organization (2021).

[34] Republic of Zambia AMRCC (2020).

[35] Kotwani A., Gandra S. (2023). Strengthening antimicrobial stewardship activities in secondary and primary public healthcare facilities in India: Insights from a qualitative study with stakeholders. Indian J Med Microbiol.

[36] Lim C., Ashley E.A., Hamers R.L., Turner P., Kesteman T., Akech S. (2021). Surveillance strategies using routine microbiology for antimicrobial resistance in low- and middle-income countries. Clin Microbiol Infect.

[37] Okeke I.N., Aboderin A.O., Egwuenu A., Underwood A., Afolayan A.O., Kekre M. (2022). Establishing a national reference laboratory for antimicrobial resistance using a whole-genome sequencing framework: Nigeria’s experience. Microbiology.

[38] Ferguson J.K., Joseph J., Kangapu S., Zoleveke H., Townell N., Duke T. (2020). Quality microbiological diagnostics and antimicrobial susceptibility testing, an essential component of antimicrobial resistance surveillance and control efforts in Pacific island nations. Western Pac Surveill Response J.

[39] Yamba K., Lukwesa-Musyani C., Samutela M.T., Kapesa C., Hang’ombe M.B., Mpabalwani E. (2023). Phenotypic and genotypic antibiotic susceptibility profiles of Gram-negative bacteria isolated from bloodstream infections at a referral hospital, Lusaka, Zambia. PLOS Glob Public Health.

[40] Mudenda S., Matafwali S.K., Malama S., Munyeme M., Yamba K., Katemangwe P. (2022). Prevalence and antimicrobial resistance patterns of Enterococcus species isolated from laying hens in Lusaka and Copperbelt provinces of Zambia: a call for AMR surveillance in the poultry sector. JAC Antimicrob Resist.

[41] Mudenda S., Malama S., Munyeme M., Matafwali S.K., Kapila P., Katemangwe P. (2023). Antimicrobial resistance profiles of Escherichia coli isolated from laying hens in Zambia: implications and significance on one health. JAC Antimicrob Resist.

[42] Yamba K., Mudenda S., Mpabalwani E., Mainda G., Mukuma M., Samutela M.T. (2024). Antibiotic prescribing patterns and carriage of antibiotic-resistant Escherichia coli and Enterococcus species in healthy individuals from selected communities in Lusaka and Ndola districts, Zambia. JAC Antimicrob Resist.

[43] Bumbangi F.N., Llarena A.-K., Skjerve E., Hang’ombe B.M., Mpundu P., Mudenda S. (2022). Evidence of community-wide spread of multi-drug resistant Escherichia coli in young children in Lusaka and Ndola Districts, Zambia. Microorganisms.

[44] Chiyangi H., Muma B., Malama S., Manyahi J., Abade A., Kwenda G. (2017). Identification and antimicrobial resistance patterns of bacterial enteropathogens from children aged 0–59 months at the University Teaching Hospital, Lusaka, Zambia: a prospective cross-sectional study. BMC Infect Dis.

[45] Republic of Zambia NAP on AMR (2017). https://www.afro.who.int/publications/multi-sectoral-national-action-plan-antimicrobial-resistance-2017-2027.

[46] Matee M., Mshana S.E., Mtebe M., Komba E.V., Moremi N., Lutamwa J. (2023). Mapping and gap analysis on antimicrobial resistance surveillance systems in Kenya, Tanzania, Uganda and Zambia. Bull Natl Res Cent.

[47] Nowbuth A., Asombang A., Tazikeng N., Makinde O., Sheets L. (2022). Antimicrobial resistance in Zambia: a systematic review. Int J Infect Dis.

[48] Mudenda S., Chilimboyi R., Matafwali S.K., Daka V., Lindizyani Mfune R., Arielle L. (2024). Hospital prescribing patterns of antibiotics in Zambia using the WHO prescribing indicators post-COVID-19 pandemic: findings and implications. JAC Antimicrob Resist.

[49] Ngoma M.T., Sitali D., Mudenda S., Mukuma M., Bumbangi F.N., Bunuma E. (2024). Community antibiotic consumption and associated factors in Lusaka district of Zambia: findings and implications for antimicrobial resistance and stewardship. JAC Antimicrob Resist.

[50] Zambia National Public Health Institute (2019). https://www.afro.who.int/publications/prioritised-activities-zambias-multi-sectoral-national-action-plan-antimicrobial.

[51] Musa K., Okoliegbe I., Abdalaziz T., Aboushady A.T., Stelling J., Gould I.M. (2023). Laboratory surveillance, quality management, and its role in addressing antimicrobial resistance in Africa: a narrative review. Antibiotics.

[52] Ackers L., Ackers-Johnson G., Welsh J., Kibombo D., Opio S. (2020). Anti-Microbial Resistance in Global Perspective.

[53] Beyene A.M., Andualem T., Dagnaw G.G., Getahun M., LeJeune J., Ferreira J.P. (2023). Situational analysis of antimicrobial resistance, laboratory capacities, surveillance systems and containment activities in Ethiopia: a new and One Health approach. One Health.

[54] Centers for Disease Control and Prevention (2020).

[55] Malania L., Wagenaar I., Karatuna O., Tambic Andrasevic A., Tsereteli D., Baidauri M. (2021). Setting up laboratory-based antimicrobial resistance surveillance in low- and middle-income countries: lessons learned from Georgia. Clin Microbiol Infect.

[56] Aldosari B., Gadi H.A., Alanazi A., Househ M. (2017). Surveying the influence of laboratory information system: An end-user perspective. Informatics Med Unlocked.

[57] Lukić V. (2017). Laboratory information system – where are we today?. J Med Biochem.

[58] Darwish R.M., Matar S.G., Snaineh A.A.A., Alsharif M.R., Yahia A.B., Mustafa H.N. (2022). Impact of antimicrobial stewardship on antibiogram, consumption and incidence of multi-drug resistance. BMC Infect Dis.

[59] Bakanidze L., Imnadze P., Perkins D. (2010). Biosafety and biosecurity as essential pillars of international health security and cross-cutting elements of biological nonproliferation. BMC Public Health.

[60] Perovic O., Yahaya A.A., Viljoen C., Ndihokubwayo J.B., Smith M., Coulibaly S.O. (2019). External quality assessment of bacterial identification and antimicrobial susceptibility testing in African national public health laboratories, 2011–2016. Trop Med Infect Dis.

[61] Iskandar K., Molinier L., Hallit S., Sartelli M., Hardcastle T.C., Haque M. (2021). Surveillance of antimicrobial resistance in low- and middle-income countries: a scattered picture. Antimicrob Resist Infect Control.

[62] Otieno P.A., Campbell S., Maley S., Obinju Arunga T., Otieno Okumu M. (2022). A systematic review of pharmacist-led antimicrobial stewardship programs in Sub-Saharan Africa. Int J Clin Pract.

[63] Basu S., Pal A., Desai P. (2005). Quality control of culture media in a microbiology laboratory. Indian J Med Microbiol.

[64] Chugh T. (2020). Diagnostic errors in clinical microbiology and antimicrobial resistance. Curr Med Res Pract.

[65] Truong W.R., Hidayat L., Bolaris M.A., Nguyen L., Yamaki J. (2021). The antibiogram: key considerations for its development and utilization. JAC Antimicrob Resist.

[66] Clinical and Laboratory Standards Institute (2020). https://unitedvrg.com/2021/05/20/m100-performance-standards-for-antimicrobial-susceptibility-testing-30th-edition-2020-pdf/.

[67] Tabah A., Cotta M.O., Garnacho-Montero J., Schouten J., Roberts J.A., Lipman J. (2016). A systematic review of the definitions, determinants, and clinical outcomes of antimicrobial de-escalation in the intensive care unit. Clin Infect Dis.

[68] Chaplain D., Asutaku B Ben, Mona M., Bulafu D., Aruhomukama D. (2022). The need to improve antimicrobial susceptibility testing capacity in Ugandan health facilities: insights from a surveillance primer. Antimicrob Resist Infect Control.

[69] Mathew P., Ranjalkar J., Chandy S.J. (2020). Challenges in implementing antimicrobial stewardship programmes at secondary level hospitals in India: an exploratory study. Front Public Heal.

[70] Baraka M.A., Alsultan H., Alsalman T., Alaithan H., Islam M.A., Alasseri A.A. (2019). Health care providers’ perceptions regarding antimicrobial stewardship programs (AMS) implementation – facilitators and challenges: a cross-sectional study in the Eastern province of Saudi Arabia. Ann Clin Microbiol Antimicrob.

[71] Dakorah M.P., Agyare E., Acolatse J.E.E., Akafity G., Stelling J., Chalker V.J. (2022). Utilising cumulative antibiogram data to enhance antibiotic stewardship capacity in the Cape Coast Teaching Hospital, Ghana. Antimicrob Resist Infect Control.

[72] Gajic I., Kabic J., Kekic D., Jovicevic M., Milenkovic M., Mitic Culafic D. (2022). Antimicrobial susceptibility testing: a comprehensive review of currently used methods. Antibiotics.

[73] Yamba K., Mpabalwani E., Nakazwe R., Mulendele E., Weldegebriel G., Mwenda J.M. (2021). The burden of invasive bacterial disease and the impact of 10-valent pneumococcal conjugate vaccine in children <5 years hospitalized for meningitis in Lusaka, Zambia, 2010–2019. J Infect Dis.

[74] Brueggemann A.B., Jansen van Rensburg M.J., Shaw D., McCarthy N.D., Jolley K.A., Maiden M.C.J. (2021). Changes in the incidence of invasive disease due to Streptococcus pneumoniae, Haemophilus influenzae, and Neisseria meningitidis during the COVID-19 pandemic in 26 countries and territories in the Invasive Respiratory Infection Surveillance Initiative: a prospective analysis of surveillancd data. Lancet Digit Health.

[75] Miller M.B., Atrzadeh F., Burnham C.A.D., Cavalieri S., Dunn J., Jones S. (2019). Clinical utility of advanced microbiology testing tools. J Clin Microbiol.

